# Supplementary material for: Continued preference for suboptimal habitat reduces bat survival with white-nose syndrome
Source: Nat Commun. 2021 Jan 8;12:166. doi: 10.1038/s41467-020-20416-5 (PMC7794521; doi:10.1038/s41467-020-20416-5)
Supplement: Supplementary file 1 — Supplementary Information [file 41467_2020_20416_MOESM1_ESM.pdf]

Continued preference for suboptimal habitat reduces bat survival with white-nose syndrome

Supplementary Discussion:

Changes in fungal loads increased with early roosting temperatures:

The best-fitting logistic growth curves for the previously published laboratory fungal growth rate data (Verant *et al.* 2012) at each average temperature treatment had the lowest RMSE when the fungal carrying capacity was set to K=0.85 (RMSE=1.23; Supplementary Figure 4). Using this K, the estimated intrinsic growth rates demonstrated the expected unimodal relationship across temperature treatments (Supplementary Figure 5). The estimated coefficients for the Logan-10 curve that best fit this relationship (with low-high credible interval cutoffs in parentheses) were a=0.72 (0.42-1.21), p=0.31 (0.17-0.82), c=5.26 (1.99-19.46), T_{opt}=14.05 (12.97-15.16), and T_{max}=21.52 (20.54-22.47). We used these 95% credible intervals to fit the Logan-10 temperature-growth curve to the changes in fungal loads that we observed on bats in the field. The best-fitting shape parameters for the Logan-10 temperature-dependent curve on bats (with low-high credible interval cutoffs in parentheses) were a=0.79 (0.44-1.19), p=0.27 (0.20-0.36), c=16.50 (10.21-19.38), and T_{opt}=13.70 (12.99-15.02)(Fig. 3). The best-fitting coefficient for the load-dependent intercept was -0.03 and the credible interval did not overlap 0 (-0.05 to -0.003), confirming that early fungal loads were a significant predictor of changes in fungal loads.

Bats shifted to colder average roosting temperatures post-invasion:

Before *P. destructans* invaded (up to and including the first year of detection), the mean early hibernation roosting temperature varied substantially amongst hibernacula (random site effect variance = 3.90), with less variation amongst sections (random section within site effect variance=0.76) or amongst microsites within sections (residual variance = 0.54)(Supplementary Figure 3). Averaging over site and section-level variation, the average population roosting temperature during pre-invasion was 8.95ºC (SE=0.02). Average population roosting temperatures were 0.7ºC higher during invasion (SE=0.05, p<0.001), when very few bats used the coldest roosts observed during pre-invasion. In contrast, average post-invasion roosting temperatures were 0.85ºC lower than pre-invasion temperatures (coefficient=-0.85, SE=0.10, p<0.001).

Temperature had contrasting effects on over summer and over winter population change

As expected based on the recapture analysis, hibernacula with the warmest early hibernation roosting temperatures during post-invasion tended to have the lowest population growth rates from November to March (Supplementary Figure 8), and thus the highest overwinter mortality. If bats were avoiding these high mortality sites, we would expect over summer population change (reflecting immigration and recruitment) to be lowest in warm sites. Instead, over summer population growth tended to be highest in warm sites (Supplementary Figure 8), as indicated by a marginally significant interaction term between the demographic season (over winter versus over summer) and temperature (interaction term=-0.14, SE=0.08, p=0.08). Given these opposite trends, the over winter population growth rate intercept was significantly higher at cold temperatures (main effect=1.43, SE=0.68, p=0.04) than the over summer population growth rate intercept (model intercept=-1.04, SE=0.68). The coefficient for the main temperature effect in the Gaussian linear model, without accounting for the interaction, was 0.10 (SE=0.07, p=0.14).

Supplementary Figures:

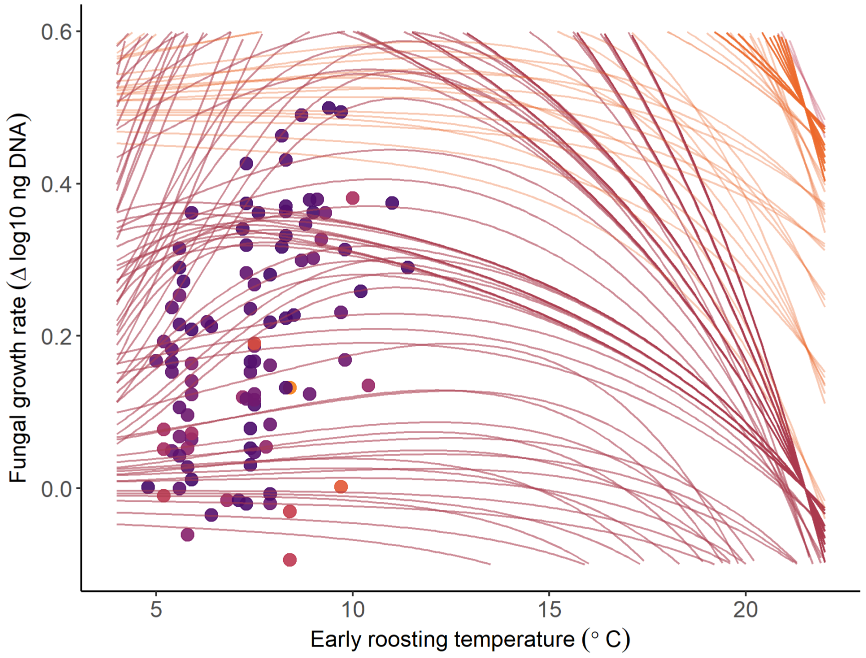

Supplementary Figure 1. **Uniform priors allowed for all possible curve shapes.** These Logan-10 temperature-dependent growth curves were plotted using parameter values sampled from the minimum, maximum, and median of the prior ranges used in the Bayesian fungal load change analysis.

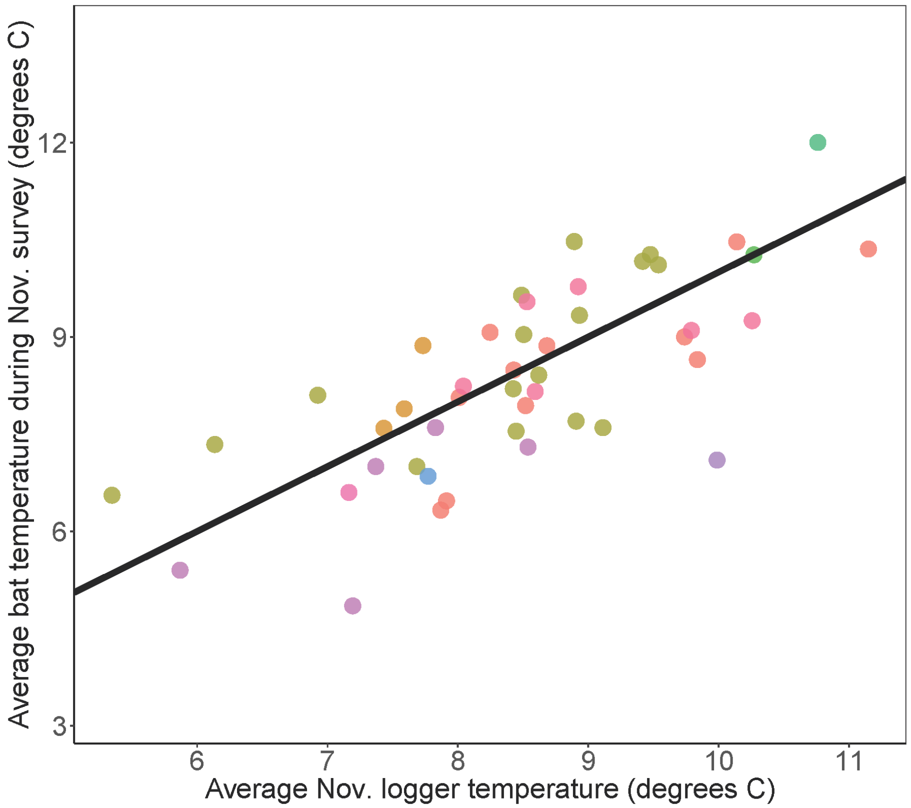

Supplementary Figure 2. **Individual bat temperature point estimates match longer-term section averages.** Bat roosting temperatures varied within sections and hibernacula (also see Supplementary Figure 3) and hibernacula temperatures varied across the hibernation season, but the point estimates for individual bats measured with the laser thermometer during early hibernation (November) surveys were consistent with the average November temperatures measured by a nearby HOBO ProV2 temperature logger. This confirms the general reliability of laser thermometer estimates. The black line shows the 1:1 line. Points with the same colors are the averages for different sections and years within the same site.

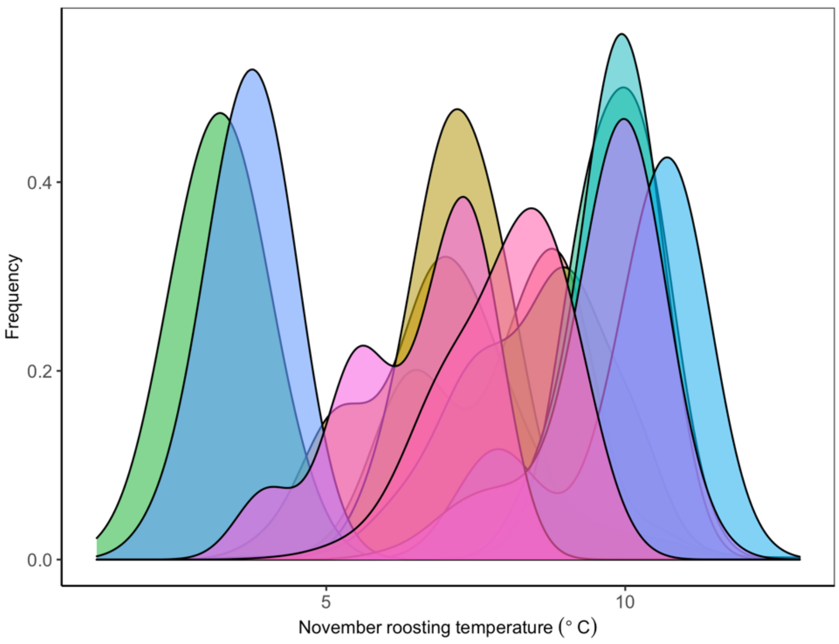

Supplementary Figure 3. **Bat roosting temperatures varied within and among hibernacula**. Density plots showing the pre-invasion distribution of early hibernation roosting temperatures where little brown bats were observed in the 12 hibernacula that were sampled before, during, and after *P. destructans* invasion. We quantified variation in early hibernation roosting temperatures within and amongst hibernacula before *P. destructans* invasion (i.e., how much variation there was for selection/learning to act on) using an intercept-only Gaussian model with random effects for site and section within site. Before invasion, bat roosting temperatures varied substantially within and between hibernacula.

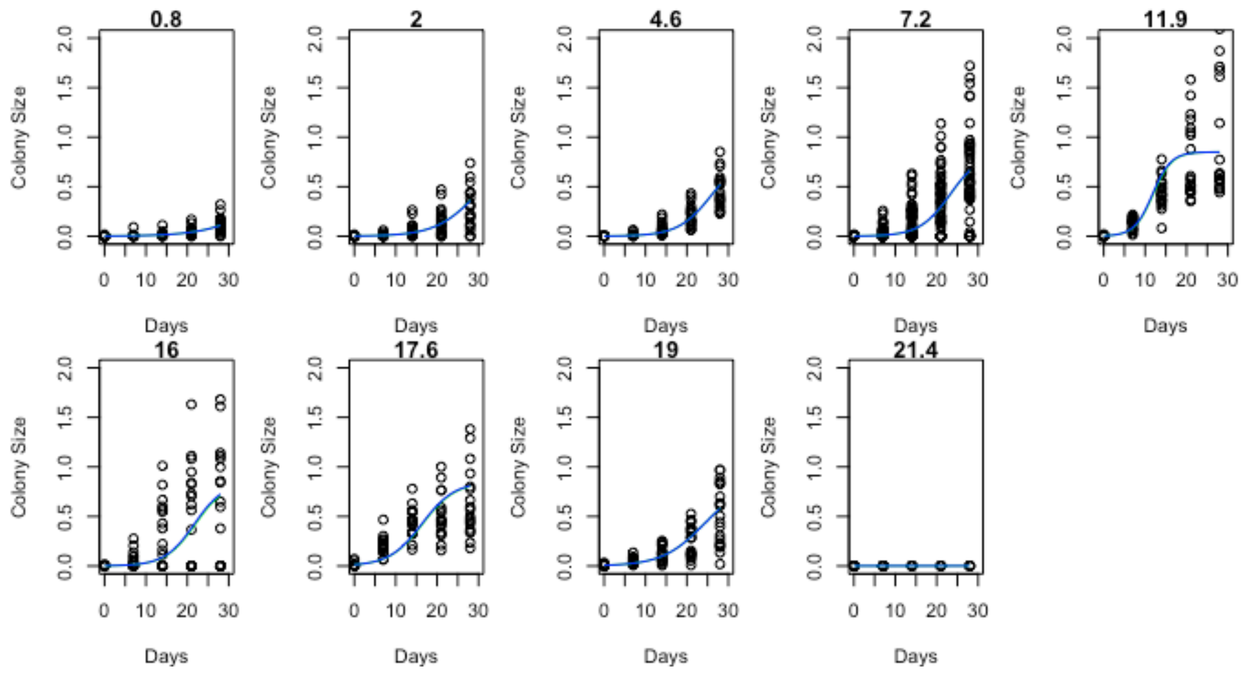

Supplementary Figure 4. **Re-calculating laboratory fungal growth rates.** The best-fitting logistic growth models for K=0.85 cm^2, fit to data from a previously published laboratory fungal growth experiment (Verant *et al.* 2012).

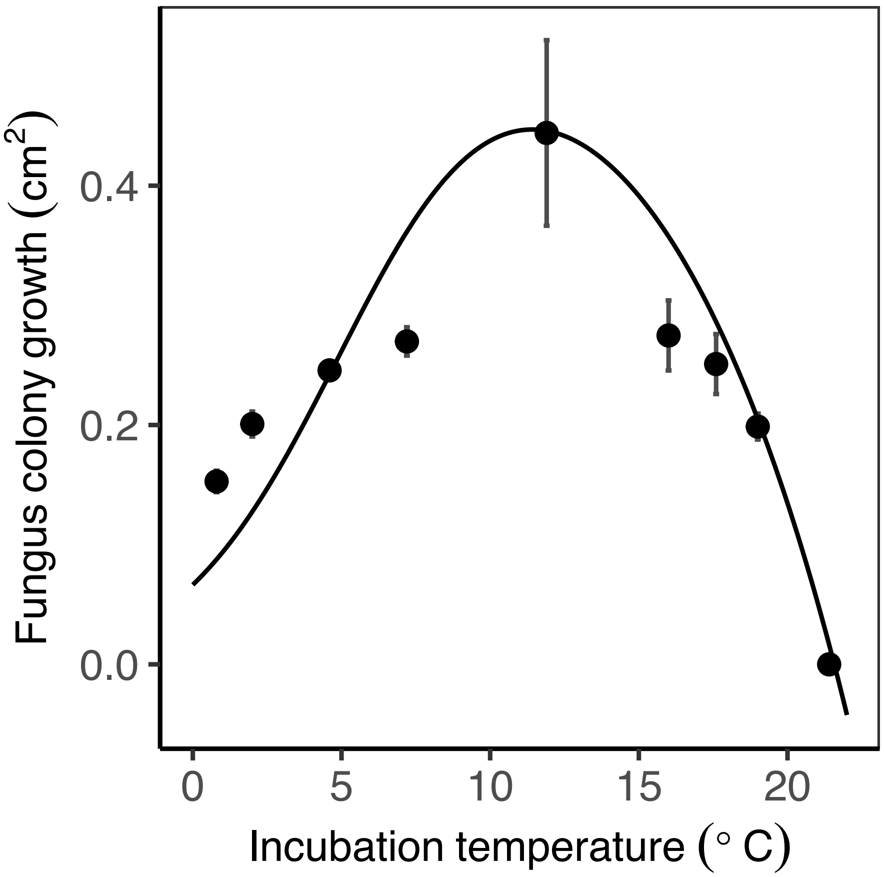

Supplementary Figure 5. **Re-quantifying the temperature-dependent laboratory fungal growth rate curve.** The best-fitting Logan-10 temperature-dependent growth curve, fit to the intrinsic growth rates estimated for each temperature treatment from a previously published laboratory fungal growth experiment (Verant *et al.* 2012).

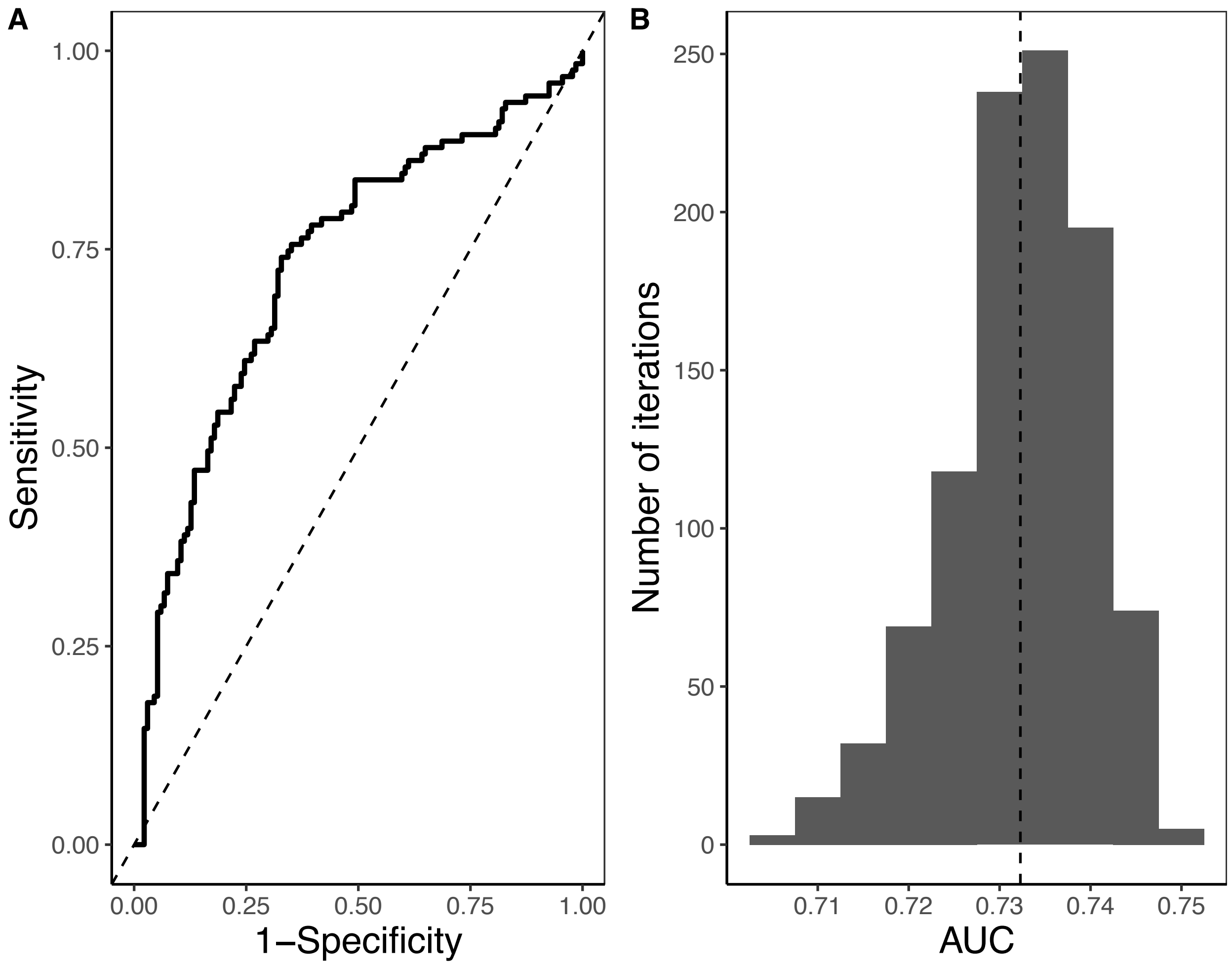

Supplementary Figure 6. **Predictive capabilities of the temperature- and load-dependent recapture model.** (A) The ROC curve for a single iteration of 5-fold cross-validation using the best-fitting logistic model describing the probability of bat recaptures. For this iteration, the AUC was 74% (95% confidence interval=67-80%). (B) The distribution of AUCs from 1000 iterations of 5-fold cross-validation. The dotted line shows the mean AUC of 73%.

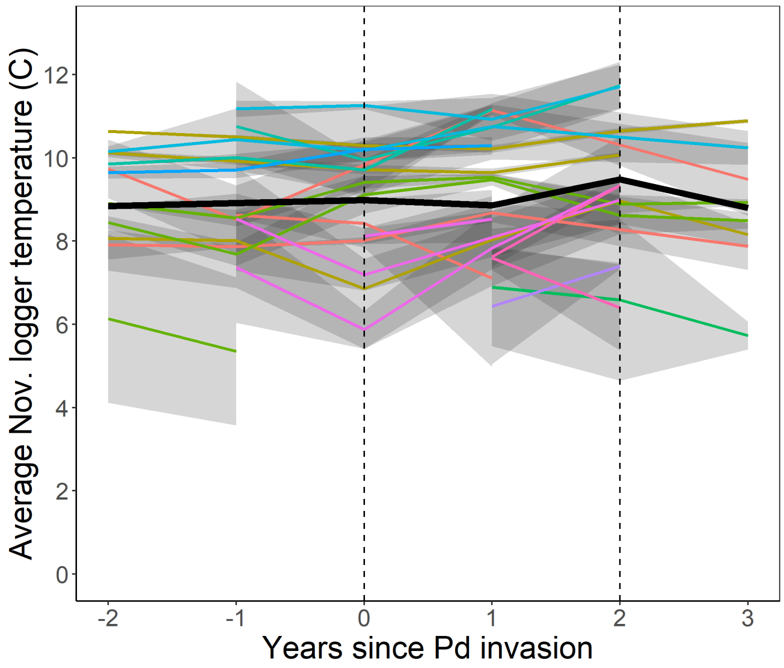

Supplementary Figure 7. **Average hibernaculum temperatures from pre- to post- invasion.** The average hibernacula temperature might have varied over time, but there was no clear trend in average early hibernation logger temperatures in sections or sites when viewed across years since the fungus invaded any given site, as not all sites were invaded in the same year. Each colored line shows the mean for a given section, colored by site (n=12 sites), bounded by a gray polygon that illustrates +/- two standard deviations. The solid black line shows the mean across all loggers in all sections and all sites, for the 12 sites where bats were sampled during pre-invasion (before year 1), invasion (years 1-2), and post invasion (year >2).

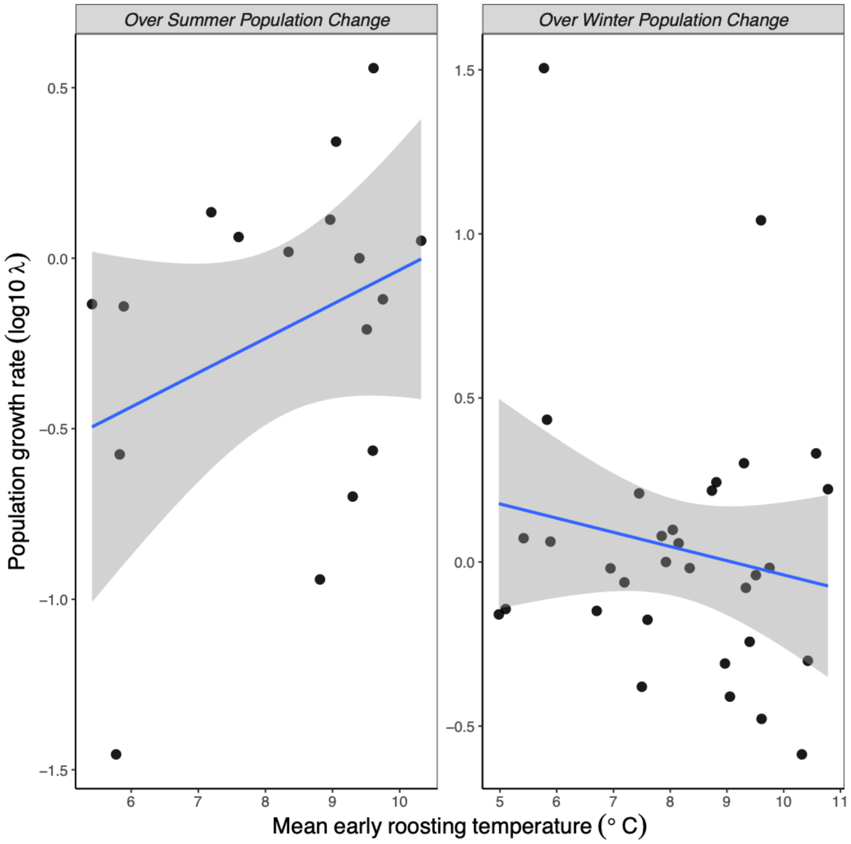

Supplementary Figure 8. **Hibernaculum-level population growth rates over summer and over winter.** During post-invasion years (>2 years after the fungus was first detected at a site), average early hibernation roosting temperatures in a given hibernaculum (n=22 sites) influenced the over summer population growth rates (immigration and recruitment between March and November) and over winter population growth rates (mortality and emigration from November to March), where higher growth rates are lower mortality rates. The estimates are given for the 22 hibernacula that we surveyed at least once post-invasion, some of which are missing over summer estimates because they were not surveyed in consecutive years, and some of which had data for more than one over winter period. The blue lines are the best-fitting Gaussian regression lines and the shaded regions are 95% confidence intervals on the model fits.
