# Supplementary material for: Continued preference for suboptimal habitat reduces bat survival with white-nose syndrome
Source: Nat Commun. 2021 Jan 8;12:166. doi: 10.1038/s41467-020-20416-5 (PMC7794521; doi:10.1038/s41467-020-20416-5)
Supplement: Supplementary file 3 — Reporting Summary [file 41467_2020_20416_MOESM3_ESM.pdf]

nature research

Corresponding author(s): Skylar Hopkins

Last updated by author(s): 11/3/2020

Reporting Summary

Nature Research wishes to improve the reproducibility of the work that we publish. This form provides structure for consistency and transparency in reporting. For further information on Nature Research policies, see our Editorial Policies and the Editorial Policy Checklist.

Statistics

For all statistical analyses, confirm that the following items are present in the figure legend, table legend, main text, or Methods section.

n/a	Confirmed	
☐	☒	The exact sample size (n) for each experimental group/condition, given as a discrete number and unit of measurement
☐	☒	A statement on whether measurements were taken from distinct samples or whether the same sample was measured repeatedly
☐	☒	The statistical test(s) used AND whether they are one- or two-sided<br>*Only common tests should be described solely by name; describe more complex techniques in the Methods section.*
☐	☒	A description of all covariates tested
☐	☒	A description of any assumptions or corrections, such as tests of normality and adjustment for multiple comparisons
☐	☒	A full description of the statistical parameters including central tendency (e.g. means) or other basic estimates (e.g. regression coefficient) AND variation (e.g. standard deviation) or associated estimates of uncertainty (e.g. confidence intervals)
☐	☒	For null hypothesis testing, the test statistic (e.g. F, t, r) with confidence intervals, effect sizes, degrees of freedom and P value noted<br>*Give P values as exact values whenever suitable.*
☐	☒	For Bayesian analysis, information on the choice of priors and Markov chain Monte Carlo settings
☐	☒	For hierarchical and complex designs, identification of the appropriate level for tests and full reporting of outcomes
☐	☒	Estimates of effect sizes (e.g. Cohen's d, Pearson's r), indicating how they were calculated

Our web collection on statistics for biologists contains articles on many of the points above.

Software and code

Policy information about availability of computer code

Data collection	No software was used to collect data in this study.
Data analysis	All script files and data needed to reproduce our analyses are published under an MIT license in the VTechData Repository (doi: 10.7294/7134-4610).

For manuscripts utilizing custom algorithms or software that are central to the research but not yet described in published literature, software must be made available to editors and reviewers. We strongly encourage code deposition in a community repository (e.g. GitHub). See the Nature Research guidelines for submitting code & software for further information.

Data

Policy information about availability of data

All manuscripts must include a data availability statement. This statement should provide the following information, where applicable:

- Accession codes, unique identifiers, or web links for publicly available datasets
- A list of figures that have associated raw data
- A description of any restrictions on data availability

All data are published in the VTechData Repository (doi: 10.7294/7134-4610)

Field-specific reporting

Ecological, evolutionary & environmental sciences study design

All studies must disclose on these points even when the disclosure is negative.

Study description	This study began in 2013, before P. destructans invaded Michigan and Wisconsin, and continued through 2020, after all Michigan and Wisconsin hibernacula were invaded by the pathogen. Throughout this period, we visited 22 hibernacula twice per winter during bat hibernation to quantify bat colony sizes, individual bat roosting temperatures, fungal loads (to which we added a constant 0.0001 before transforming to the log10 scale), and recapture probabilities. We sampled bats during early hibernation in November, when more than 95% of swarm activity was expected to be finished, and again during late hibernation in March, when less than 1% of spring emergence activity was expected to have begun. For each sampling event, we counted all bats of all species within the site. We focused our analyses on the little brown bat (Myotis lucifugus). We divided bat counts by sections within the hibernaculum (i.e., "rooms") that potentially varied in microclimate, and we used HOBO Pro v2 data loggers to continuously record temperatures every three hours in these sections. After counting all bats, we haphazardly sampled 20-25 individual little brown bats stratified across sections and roughly in proportion to the number of bats in each section.
Research sample	We focused our analyses on the species that was most common before P. destructans invasion, the little brown bat (Myotis lucifugus), because it provided adequate sample sizes and had been substantially impacted by the disease. We sampled adult bats of both sexes.
Sampling strategy	We divided bat counts by natural sections within the hibernaculum (i.e., "rooms") that potentially varied in microclimate, and we used HOBO Pro v2 data loggers to continuously record temperatures every three hours in these sections. After counting all bats, we haphazardly sampled 20-25 individual little brown bats stratified across sections and roughly in proportion to the number of bats in each section. We chose to sample up to 25 bats per site per survey because this would allow us to detect the fungus if at least 4% of bats were infected (1/25). We did not sample more than 25 individuals per site because we wanted to limit disturbance to hibernating bats.
Data collection	For each sampled bat, we used a Fluke 62 MAX IR laser thermometer to quantify the temperature of the substrate directly adjacent to the bat (<1 cm)(i.e., the "roosting temperature"). We then used a previously developed protocol to collect a standardized swab of each bat's forearm and muzzle, which we stored in RNAlater until we could quantify fungal loads using qPCR (Langwig et al. 2015a). Finally, we banded as many swabbed bats as possible with an aluminum band (2.9 mm; Porzana Ltd., Icklesham, E. Sussex, U.K.), so that they could be re-sighted and individually-identified during subsequent visits. One team member trained in the recording procedure recorded the data during each survey.
Timing and spatial scale	Beginning in 2013, before P. destructans invaded Michigan and Wisconsin, and continuing through present (2020), when almost all Michigan and Wisconsin hibernacula have been invaded, we visited 22 hibernacula twice per year during bat hibernation (October to April). Individual bats could be touching, separated by millimeters, or separated by tens of meters with a given hibernaculum.
Data exclusions	We only used bats that were infected during our November samples in our analyses of recapture rates and fungal growth rates, because we wanted to know how temperature affected disease outcomes after infection. We excluded bats that were uninfected in November because if they had been infected in March, we would not have known how long they had been infected for. The decision to exclude uninfected bats was pre-established before we ran our analyses. For the distribution shift analysis, we only included sites that were sampled during all three invasion periods, so as not to skew the mean roosting temperature in any given period by adding new sites to the analysis. This decision was pre-established before we ran the analysis.
Reproducibility	In the fungal load change analysis, each infected, recaptured bat was considered an independent replicate (N=123), and we used a standard reproducible method to quantify fungal loads. We used simple correlation tests and a Logan-10 growth curve to confirm that there was a relationship between fungal load change and temperature. The code to reproduce these analyses is available in the repository in the Code Availability section.<br>In the recapture rate analysis, each infected, banded bat was considered an independent replicate (N=259). We used five-fold cross-validation to show that our logistic regression model could predict whether a bat would be recaptured with 73% accuracy. The code to reproduce this analysis is available in the repository in the Code Availability section.<br>In the distribution shift analysis, each counted bat was considered an independent replicate (N=8473). The code to reproduce this analysis is available in the repository in the Code Availability section.
Randomization	As described above, we stratified sampling within hibernacula by section/room, and then haphazardly sampled bats within sections/rooms.
Blinding	NA - researchers are blind to infection load and microtemperatures until after they take the measurements, so this did not affect our sampling design.

Did the study involve field work? ☒ Yes ☐ No

Field work, collection and transport

Field conditions	We visited mines during the winter, when bats were hibernating. Temperatures varied within and between mines, as quantified in our study. We did not quantify weather conditions outside of the mines, because this was not relevant to our study.
Location	We surveyed 22 mine hibernacula in Michigan and Wisconsin. Due to the sensitive nature of mines and bat conservation concerns, precise locations are not provided.
Access & import/export	All sites and sampling procedures were covered by the appropriate, annually-renewed state permits for bats (Michigan Permit SC 1651, Wisconsin permit 882), Virginia Tech IACUC protocol #17-180, and University of California, Santa Cruz IACUC protocol

	Kilpm1705. We followed field hygiene and decontamination protocols in accordance with United States Fish & Wildlife Service.
Disturbance	We followed field hygiene and decontamination protocols in accordance with United States Fish & Wildlife Service to avoid spreading the pathogen among bats or hibernacula.

Reporting for specific materials, systems and methods

We require information from authors about some types of materials, experimental systems and methods used in many studies. Here, indicate whether each material, system or method listed is relevant to your study. If you are not sure if a list item applies to your research, read the appropriate section before selecting a response.

Materials & experimental systems

n/a	Involved in the study
☒	☐ Antibodies
☒	☐ Eukaryotic cell lines
☒	☐ Palaeontology and archaeology
☐	☒ Animals and other organisms
☒	☐ Human research participants
☒	☐ Clinical data
☒	☐ Dual use research of concern

Methods

n/a	Involved in the study
☒	☐ ChIP-seq
☒	☐ Flow cytometry
☒	☐ MRI-based neuroimaging

Animals and other organisms

Policy information about studies involving animals; ARRIVE guidelines recommended for reporting animal research

Laboratory animals	No laboratory animals were used in this study.
Wild animals	Little brown bats (male and female) were swabbed, banded, and released immediately.
Field-collected samples	No field collected samples were used in this studied besides the wild bats described above.
Ethics oversight	All sites and sampling procedures were covered by the appropriate state and federal permits for bats, Virginia Tech IACUC protocol #17-180, and University of California, Santa Cruz IACUC protocol Kilpm1705.

Note that full information on the approval of the study protocol must also be provided in the manuscript.
